# GP delivered brief weight loss advice: associations between in-consultation behaviour change techniques and patient weight loss in recorded primary care discussions

**DOI:** 10.1080/21642850.2023.2213751

**Published:** 2023-05-20

**Authors:** Eleanor Ayre, Joseph J. Lee, Kerstin Frie, Paul Aveyard, Charlotte V. A. Albury

**Affiliations:** Department of Primary Care Health Sciences, University of Oxford, Oxford, UK

**Keywords:** Obesity, primary care, behaviour change, family practice, weight loss

## Abstract

**Background:**

Primary care clinicians are encouraged to intervene opportunistically, offering weight-loss advice to people living with obesity. The BWeL trial showed patients receiving brief weight-loss advice from their general practitioner lost weight at one year follow-up. We examined the behaviour change techniques (BCTs) clinicians used to identify which BCTs are associated with this weight loss.

**Methods:**

We coded 224 audio recorded interventions from the BWeL trial using the behavioural change techniques version one taxonomy (BCTTv1) and the ‘refined taxonomy of behaviour change techniques to help people change their physical activity and healthy eating behaviours’ (CALOR-RE taxonomy). Linear and logistic regressions were performed to analyse associations between behaviour change techniques used in these taxonomies and patient weight loss.

**Results:**

Mean intervention length was 86 s*.* We identified 28 different BCTs BCTTv1 and 22 from CALOR-RE. No BCTs or BCT domains were associated with mean weight loss at 12 months, loss of 5% bodyweight, or action taken at 3 months. The BCT ‘Feedback on outcomes of behaviour (future)’ was associated with an increased likelihood that the patient reported taking action to lose weight by 12 months (OR = 6.10, 95%CI = 1.20, 31.0).

**Conclusion:**

Although we found no evidence to support the use of particular BCTs, our results suggest that it is the brief intervention itself, rather than specific content, which may motivate weight loss. This can support clinicians to confidently intervene without needing complex training. Offering follow-up appointments can support positive changes to health behaviours, even if these are not associated with weight loss.

## Introduction

Obesity is a major cause of chronic illness and premature death worldwide, and its prevalence is rising (Agha & Agha, 2017; WHO, 2015). The longevity and frequency of the patient–clinician relationship in primary care means that clinicians in this setting are well placed to intervene to provide advice, and international guidelines state that primary care doctors (GPs) should opportunistically discuss weight loss with patients with obesity (Brauer et al., 2015; Moyer, 2012; NICE, 2014a). However, studies of consultation notes have shown that GPs do this infrequently, especially when weight is unrelated to the presenting complaint (Noordman et al., 2010). Healthcare professionals cite many reasons why they do not intervene, such as lack of time and training, lack of confidence that advice is effective, as well as worries of jeopardising the patient–doctor relationship (Alexander et al., 2007; Gunther et al., 2012; Nolan et al., 2012; Tsiga et al., 2013). Patients, however, report that they welcome such discussions if they are appropriately delivered, and emphasise the importance of the clinician’s communication in ensuring discussions are well received (Albury et al., 2020a Malterud & Ulriksen, 2011; Merrill & Grassley, 2008;). Patients state that these discussions can motivate them to lose weight, but can also demotivate them (Brown et al., 2006; Ward et al., 2009). Understanding how GPs and patients talk about weight in ways which are appropriate and motivate weight loss could support clinicians and patients to have more effective conversations. Effective GP initiated advice to lose weight could prevent the development of chronic diseases in people with obesity (Appel et al., 2011; Ross et al., 2008).

In this paper, we analyse audio-recordings of GP consultations which were part of the Brief interventions for Weight Loss (BWeL) trial (Aveyard et al., 2016). GPs in the BWeL trial delivered very brief (30 s) opportunistic advice to patients with obesity at the end of a typical consultation to unselected patients with obesity, of whom 83% agreed to participate in the trial. In the arm where GPs gave advice to lose weight, there was a weight loss of 1.04 kg (SD 5.50 kg) at 12 months for people with obesity (BMI of ≥30 kg/m²) (Aveyard et al., 2016). A meta-analysis of general population cohorts indicates that the average weight loss of a patient in the general population with a BMI of ≥30 kg/m² over a year is about 300 g (Whitlock et al., 2002). However, in the arm of the BWeL trial where GPs gave advice to lose weight, there was a weight loss of 1.04 kg (SD 5.50 kg) at 12 months for people with obesity (BMI of ≥30 kg/m²) highlighting the motivational effect of even very brief advice from a GP. Even so, there was variability in patients’ weight trajectories following the consultation, suggesting that some patients were demotivated by the consultation. The trial also showed that the brief interventions were acceptable to patients, with 1530 (81%) of the 1855 included in the analysis of appropriateness describing the intervention as both appropriate and helpful, whereas only four (0.2%) individuals found the intervention inappropriate and unhelpful. Here we analyse the consultations in which people were given advice to lose weight to assess how they were delivered and whether differences in the way they were delivered are associated with a greater likelihood of patients taking action on their weight and losing weight.

Behavioural change techniques (BCTs) are ‘observable, replicable, and irreducible’ (Michie et al., 2013) components of interventions which aim to change the patient’s behaviour, described as the ‘active ingredients’ of the intervention (Michie et al., 2013). The v1 behavioural change taxonomy consists of 93 techniques grouped under 16 categories (called ‘domains’), where BCTs within each domain share a similar mechanism of change. Whilst the v1 taxonomy is designed to be broadly applicable to interventions aimed to change most types of behaviour, the CALO-RE taxonomy is a ‘refined taxonomy of behaviour change techniques to help people change their physical activity and healthy eating behaviours’ (Michie et al., 2011) and consists of 40 BCTs. Whilst the BCT v1 taxonomy provides more BCTs, and captures breadth, the CALOR-E taxonomy allows finer-grained coding in some areas, and offers more topic specific techniques in depth. For example, CALO-RE introduces BCTs such as ‘fear arousal’, which studies have shown have a negative impact on motivation to lose weight (Gray et al., 2011), and ‘motivational interviewing’ which is a technique focusing on the patients reasons and motivations for losing weight (Michalopoulou et al., 2022). CALO-RE also breaks down ‘information on health consequences’ which is a single BCT in the v1 taxonomy into ‘general’ and ‘individual’. Both taxonomies have been used previously in weight loss literature (Bourhill et al., 2021; Cradock et al., 2017; French et al., 2014; Hartmann-Boyce et al., 2014). The BCTs used by GPs when giving very brief advice as part of the BWeL trial may have motivated (or demotivated) weight loss. Finding out which BCTs were used, and with what effect, could indicate the ‘active ingredients’ of effective GP-initiated advice to lose weight and assist GPs in knowing what to say that may support weight loss.

Systematic reviews show mixed evidence about whether BCTs affect weight loss (Hartmann-Boyce et al., 2014; Michie et al., 2009), as well as which BCTs work best at motivating weight loss behaviours (Greaves et al., 2011). However, these studies largely code reports of BCTs used in intervention design, rather than in recorded conversations, and rarely focus on very brief interventions. This is important because there is often a gap between what is intended to be delivered and what actually is delivered. This will tend to cloud associations between BCTs and behaviour change, biasing associations towards the null. For example, one study examining fidelity of delivery of BCTs in a physical activity intervention showed that adherence differed between facilitators delivering the trial, and also decreased over time (Hardeman et al., 2008). The audio recorded data from the BWeL trial, however, provides a good opportunity to examine BCTs actually used during delivery of very brief advice.

Brief advice on behaviour change can promote the outcome in two ways: motivating action in people who would not otherwise have acted, and sustaining action through increased motivation in those who take action. There is evidence from a trial that brief advice for smoking cessation works in both ways, prompting quit attempts and boosting the success of attempts that occur; though prompting an attempt was the dominant mechanism (Russell et al., 1979). There is currently mixed evidence on whether using more BCTs in a consultation leads to better outcomes (Dombrowski et al., 2012; Hankonen et al., 2014). Whilst existing work focusses on reports of BCTs used in interventions, our focus on real recordings can contribute to this current area of uncertainty. In this paper, we used two BCT taxonomies (v1 and CALOR-E) to code audio recorded intervention data from the BWeL trial. We aimed to identify whether the use of BCTs in GP-delivered opportunistic weight loss advice motivates patients to take action and whether that results in weight loss. The objectives were:

1. To assess whether individual BCTs, or BCT domains used by GPs when giving weight loss advice, were associated with weight loss at 12 months.
2. Losing ≥5% of one’s bodyweight has been identified as clinically significant (Blackburn, 1995; Brown et al., 2016), so we assessed whether BCTs or BCT domains were associated with patients losing ≥5% of their bodyweight.
3. BCTs used by clinicians may not have resulted in weight loss, but could have successfully supported people to take action on their weight, for example making changes to physical activity or dietary behaviours. Therefore, our third objective was to identify whether any BCTs or BCT domains were associated with patients reporting having taken action on their weight at 3 and 12 months.
4. Information about the most effective amount of BCTs used would allow clinicians to integrate these into their appointments. Our fourth objective was therefore to find out if the number of individual BCTs used was associated with patient weight loss, or action on their weight.

## Materials and methods

### Data sourcing and processing

Recorded consultation data were collected between 4 June 2013 and 23 December 2014 as part of the BWeL trial. BWeL was a parallel two-armed randomised trial, where patients with a BMI of ≥30 kg/m² and raised body fat percentage were randomised to either receive very brief GP-delivered weight loss advice or the offer of a free 12-week referral to a community weight management group. Participating GPs received video-mediated training and, in the advice-giving arm of the trial, this training asked GPs to describe how health would improve through weight loss, giving a specific example that could be pertinent to the patient, and to use their own words to achieve this. This is classified as the BCT ‘information about health consequences’.

At the beginning of the trial, the participants were weighed using the Tanita SC-240MA body composition analyser, and their height was also measured. BMI was calculated from these measurements. Participants were then telephoned after three months, where they self-reported weight loss, and then were seen by a researcher at 12 months, where they were weighed again with the same machine and had their BMI calculated. Immediately after the consultation, participants rated the intervention on five-point Likert scales from ‘very unhelpful’ to ‘very helpful’, and ‘very inappropriate’ to ‘very appropriate’. At both 3 and 12 months, the researchers determined whether participants had tried to lose weight and how they had done this. This was done through self-reporting by the participants, with BWeL researchers filling out a form which characterised this action into (1) visiting the GP to discuss weight in more detail, (2) attending a weight management programme, (3) increasing exercise, (4) changing eating habits and (5) using pharmacotherapy.

### Recording processes

**Figure 1.**
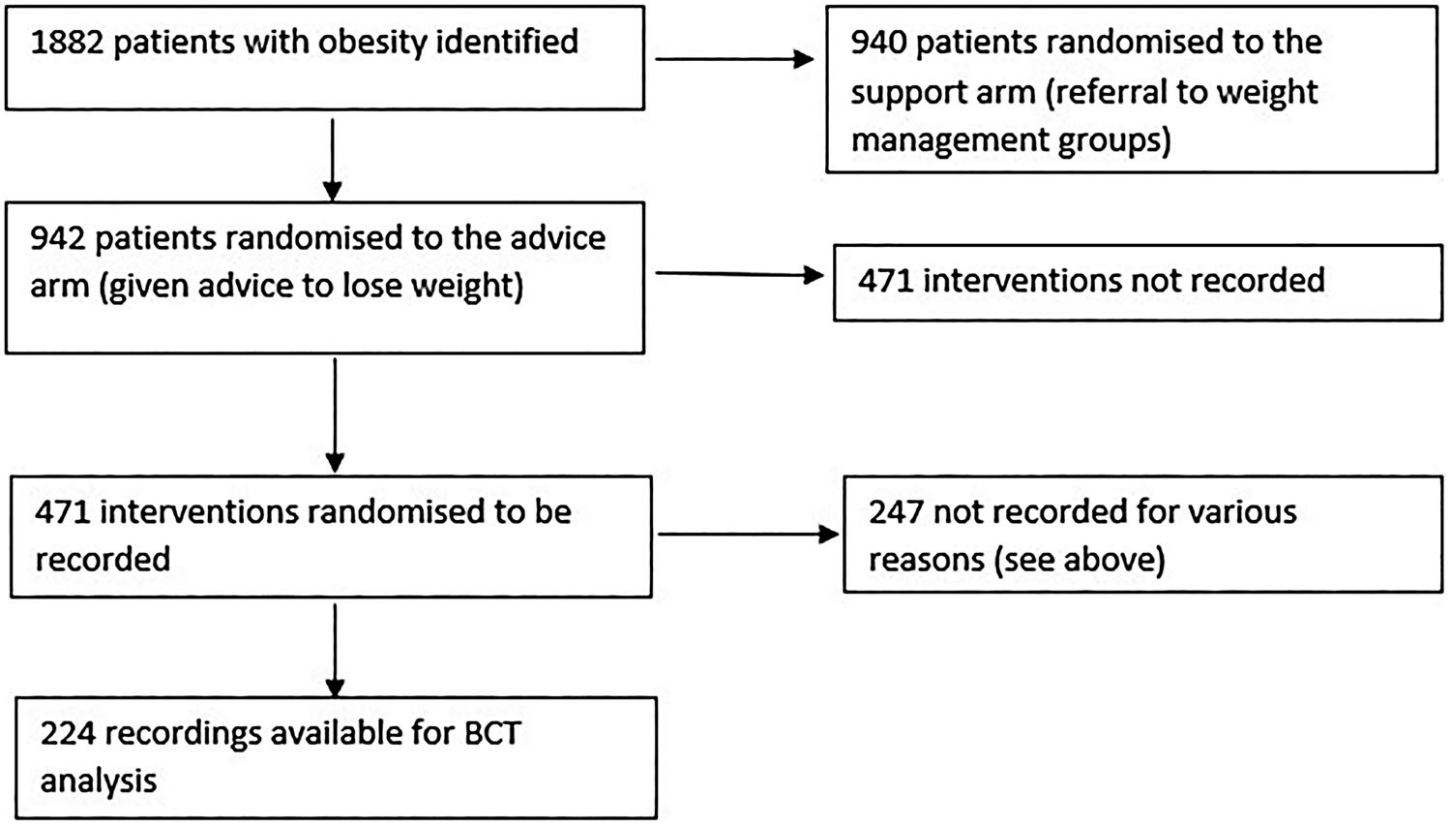
How recordings were obtained for BCT analysis.

Of 942 patients who received brief advice half were randomised to have their intervention recorded. However, some doctors deviated from protocol, offering referral rather than advice; some participants did not consent to be recorded; some recordings were not downloaded from the recording device; and some recordings were unavailable due to technical issues. This meant we had 224 recordings available for analysis (Figure 1). Recordings were collected on a hand-held recorder which was visible to both doctor and patient, and the doctor pressed record once the patient’s presenting concern had been discussed, and they were about to initiate weight loss advice. Recorded data were transcribed verbatim, and the recordings and transcripts were stored on secure hard-drives at the University of Oxford. The BWeL trial is registered with the ISRCTN Registry, number ISRCTN26563137 and details are available in the trial report (Aveyard et al., 2016). Ethical approval was granted by the NHS Research Ethics Service (reference: 13/SC/0028).

### Coding

We coded the data using both the v1 behavioural change taxonomy and the CALO-RE taxonomy. Authors 3 and 5 had been trained in using the BCT taxonomies prior to this study, and author 1 took part in online training before commencement. Authors 1 and 5 initially coded 12 transcripts independently using first the original v1 and then CALO-RE taxonomy, with reference to Michie’s BCT coding guides (Michie et al., 2011; Michie et al., 2013). The coders then met to discuss their coding, and agreed on project-specific definitions for each technique used in the BWeL transcripts, and recorded these in a coder’s handbook (as shown in Supplementary Tables 4 and 5). Authors 1 and 5 then coded another 10 transcripts to test and refine the coder’s handbook. Any discrepancies were resolved through discussion, involvement of a third coder, author 3 or referral to Michie’s coding guide. The remaining transcripts were coded by author 1. During this time, author 5 coded another 10 transcripts at random comparing and discussing results with author 1, to mitigate coder drift. When author 1 identified a new technique not yet in the coder’s handbook she discussed with author 3 and author 5 and referred to Michie’s guide to agree on a coding rule, and a description for the coder’s handbook. This handbook was updated throughout the process, and all coders agreed on content.

Existing studies have adapted BCT definitions for contextual relevance (Schichtel et al., 2019; Tate et al., 2016) and we took that approach here. From the v1 taxonomy we adapted the definition of ‘review outcome goals’ from ‘Review outcome goal(s) jointly with the person and consider modifying goal(s) in light of achievement’ (Michie et al., 2013) to ‘Review and provide feedback on previous weight change’ (as shown in Supplementary Table S4). The definition of ‘Feedback on outcomes of behaviour’ was adapted from ‘Monitor and provide feedback on the outcome of performance of the behaviour’ (Michie et al., 2013) to ‘Plan to provide feedback on the outcome of behaviour (weight) in the future usually through setting up another appointment in a specific time frame’ (as shown in Supplementary Table S4). ‘Information about evidence base’ was added, as when we were coding we noted that many doctors when giving information about health consequences stated ‘evidence shows that … ’ and together we decided that this may have a different effect on the patients’ behaviour than what we were coding for ‘information about health consequences’.

In the CALO-RE taxonomy the definition of ‘feedback on performance’ was adapted from ‘providing the participant with data about their own recorded behaviour or commenting on a person’s behavioural performance’ (Michie et al., 2011) to ‘providing the participant with information on their recent behavioural outcomes to motivate behaviour’ (see Supplementary Table S5). The definition for ‘Review outcome goals’ was adapted from ‘involves a review or analysis of the extent to which previously set outcome goals (e.g. to reduce blood pressure or lose/maintain weight) were achieved’ (Michie et al., 2011) to ‘plan to review patient performance/ outcomes in a specific timeframe’ (see Supplementary Table S5). These changes were made to relate to past weight loss and future follow up. This was because future follow up was offered by GPs relatively often, and they also commonly focussed on previous weight loss efforts, and we decided this could possibly affect a patients’ motivation to lose weight.

For each transcript BCTs were coded as 1 (present) or 0 (absent). We first coded all transcripts using the v1 taxonomy first and upon completion coded each a second time using the CALO-RE taxonomy. All coders were blinded to patient outcome data.

### Statistical analysis

We prespecified an analysis protocol (Ayre et al., 2019). We identified potential confounders including age, sex, baseline weight and IMD score. The Index of Multiple Deprivation (IMD) score is a UK government summary measure of levels of deprivation, ranking each small area of England from 1 (least deprived) to 32,844 (most deprived) (Government efCaL, 2015). We restricted analyses of BCT domains to where one or more domain components were used in more than 10% of consultations. The sample size of 224 cases allowed detection of an effect size of 1.22 kg weight change with power of 80% and alpha (or *p* value) of 5%.

BCTs were examined for pairwise collinearity (Pearson, 1896). Where necessary, highly correlated BCTs were grouped or omitted. Those with missing data for the 12-month follow up had baseline weight carried forward (therefore imputing no weight change), and data was reported as missing for whether they took action.

We assessed the association between BCTs and outcomes with linear and logistic regression for continuous and binary outcomes respectively. These analyses were repeated for each taxonomy separately. For all objectives we performed univariable and multivariable regression, which was adjusted for age, sex, baseline weight, and IMD score for all objectives. Because the numbers were small, we used fixed effects models but, as sensitivity analyses, we performed mixed effects regression allowing for clustering at the GP level.

For patient-reported appropriateness and helpfulness after the consultation, we classified >3 as ‘appropriate’ or ‘helpful’.

When assessing relationships with subsequent action (reported as (1) visiting the GP to discuss weight in more detail, (2) attending a weight management programme, (3) increasing exercise, (4) changed eating habits and (5) the use of pharmacotherapy), we excluded ‘visiting the GP to discuss weight in more detail’, as many GPs in these recordings asked patients to book an appointment to discuss weight further.

## Results

The 224 participants were recruited from 35 practices, and consulted with 82 participating GPs. Participants had a mean age of 56.67 years (Table 1), and a mean IMD score of 14.65 (national average, 21.70). The participants had a mean baseline weight of 99.2 kg, and of the 224 participants 129 were female and 95 were male. There were 46 missing 12-month weights, 21 in men, 25 in women. The mean intervention length was 86 s (range 7–532 s).

**Table 1.** Baseline characteristics.

Characteristic		*N* (%) or mean (*SD*)
Participants		224
Sex	Female	129 (57%)
	Male	96 (43%)
Baseline weight kg	Female	92.29 (13.43)
	Male	108.56 (19.58)
Age		56.67 (15.97)
IMD score		14.65 (10.84)

N = number.

Of 225 participants three (1.3%) reported the intervention was unhelpful and not appropriate; 23 (10.2%) reported it was appropriate but not helpful; seven (3.1%) reported it was helpful but not appropriate; and 192 (85.3%) reported it was both appropriate and helpful.

We initially identified 28 different BCTs from the v1 taxonomy, but ‘instruction on how to perform behaviour’ and ‘credible source’ were highly collinear and therefore were combined. We identified 22 different BCTs used from the CALO-RE taxonomy.

Eight BCTs from the v1 taxonomy and nine BCTs from the CALO-RE taxonomy were used ≥10% of the time, with 11 unique BCTs across the two taxonomies. On average, three (*SD* 1.93) BCTs were used per consultation. The most common BCTs from v1 were: Information about health consequences (*n* = 220), social support (practical) (*n* = 51); and review behaviour goals (*n* = 49). The most common BCTs from CALO-RE were: provide information on consequences of behaviour in general (*n* = 163); provide information on consequences of behaviour to the individual (*n* = 110); and fear arousal (*n* = 59). Further detail on most frequent BCTs, Supplementary Table S1.

BWeL training in brief advice included only one BCT, ‘information about health consequences’. In 220/224 recordings, the GP delivering the intervention used ‘information about health consequences’ showing 98% fidelity. Therefore, we excluded this BCT from analysis, as there were only four consultations where it did not occur.

### Objective 1

*Association between BCTs and weight loss*: Eight different BCTs from the v1 taxonomy and nine from the CALOR-E taxonomy occurred in more than 10% of consultations and were used in the analysis. In 17 analyses, there was no evidence of association between the BCTs and weight change. However, people who received a brief intervention that involved ‘instruction on how to perform behaviour’ gained more weight on average (adjusted mean weight change) was 2.53 kg (95% CI = 0.10–4.96, *p* = 0.04) than people receiving an intervention without this BCT, meaning that they gained around 1.5 kg at 12 months, given the overall mean was a weight loss of 1.04 kg (Tables 2 and 3).

**Table 2.** v1 taxonomy BCTs and mean weight loss at 12 months.

v1 Taxonomy		Univariable analysis of BCTs	Multivariable analysis of BCTs*	Domain name	Domain adjusted*
BCT	N (%)	Mean weight change kg (95% CI, *p* value)	Mean weight change kg (95% CI, *P* value)		Domain Coefficient (95% CI) (*p* value)
1.1. Goal setting (behaviour)	32 (14%)	0.31 (−1.90 to 2.52, 0.78)	−0.85 (−3.56 to 1.86, 0.53)	Goals and planning	−0.15 (−2.01 to 1.71, 0.87)
1.2. Problem solving	24 (10%)	0.45 (−2.05 to 2.94, 0.73)	−0.92 (−3.98 to 2.13, 0.55)		
1.5. Review behaviour goal(s)	49 (22%)	0.94 (−0.93 to 2.80, 0.32)	1.04 (−1.07 to 3.16, 0.33)		
2.6. Biofeedback	30 (13%)	−0.39 (−2.65 to 1.88, 0.74)	−1.24 (−3.62 to 1.13, 0.30)	Feedback and monitoring	0.38 (−1.50 to 2.26, 0.69)
2.7. Feedback on outcome(s) of behaviour future	35 (14%)	1.85 (−0.27 to 3.96, 0.09)	1.93 (−0.28 to 4.14, 0.09)		
3.2. Social support (practical)	51 (23%)	−0.19 (−2.03 to 1.65, 0.84)	−0.29 (−2.19 to 1.61, 0.76)		−0.48 (−2.35 to 1.40, 0.62)
Instruction on how to perform the behaviour and credible source	35 (16%)	2.21 (0.11 to 4.32, 0.04**)	2.53 (0.10 to 4.96, 0.04**)		2.17 (−1.6 to 4.51, 0.068)
10.4. Social reward	29 (13%)	0.15 (−2.15 to 2.45, 0.90)	−0.47 (−2.93 to 2.00, 0.71)		−0.32 (−2.79 to 2.15, 0.80)

BCT = Behavioural Change Technique, 95% CI = 95% confidence interval, *N* = number. *Adjusted for other BCTs or domains, age, sex, baseline weight and IMD score. **p < 0.05.

**Table 3.** CALO-RE taxonomy BCTs and mean weight loss at 12 months.

CALO-RE		Univariable	Multivariable
BCT	*N* (%)	Mean weight loss kg (95% CI, *P* value)	Mean weight loss kg (95% CI, *P* value)
Provide information on consequences of behaviour in general	163 (73%)	−0.10 (−1.82 to 1.63, 0.91)	−0.34 (−2.69 to 2.02, 0.78)
Provide information on consequences of behaviour to the individual	110 (49%)	−0.27 (−1.81 to 1.28, 0.73)	−0.73 (−2.76 to 1.30, 0.48)
Goal setting behaviour	32 (14%)	0.31 (−1.90 to 2.52, 0.78)	−0.76 (−3.49 to 1.97, 0.59)
Barrier identification/ Problem solving	22 (10%)	0.43 (−2.16 to 3.02, 0.74)	−0.84 (−0.39 to 2.22, 0.59)
Prompt review of behavioural goals	49 (22%)	1.35 (−0.51 to 3.21, 0.15)	1.20 (−0.84 to 3.23, 0.25)
Prompt review of outcome goals (future)	35 (16%)	1.85 (−0.27 to 3.96, 0.09)	1.74 (−0.53 to 4.01, 0.13)
Fear arousal	59 (26%)	−1.05 (−2.80 to 0.69, 0.24)	−0.96 (−2.81 to 0.90, 0.31)
Informing how to do task	35 (16%)	2.21 (0.11 to 4.32, 0.04**)	2.43 (−0.07 to 4.94, 0.06)
Plan social support	50 (22%)	−0.29 (−2.15 to 1.56, 0.75)	−0.51 (−2.44 to 1.41, 0.60)

BCT = Behavioural change technique, 95% CI = 95% confidence interval, *N* = number. **p < 0.05.

There were two domains used in the analysis of the v1 taxonomy, goals and planning and feedback and monitoring, with no evidence of an association between domain use and weight change.

### Objective 2

*Losing 5% bodyweight*: 17% of people lost 5% of their baseline weight. There was no evidence that consultations that included any of the 17 BCTs that were used in at least 10% of consultations nor either domain that was used this frequently were associated with a greater likelihood (Tables 4 and 5).

**Table 4.** v1 Behavioural Change Techniques and their impact on 5% bodyweight loss.

v1		Univariable analysis of BCTs	Multivariable analysis of BCTs	Domain name	Multivariable analysis of domain
BCT	*N* (%)	OR (95% CI, *P* value)	OR (95% CI, *P* value)		Domain OR (95% CI, *P* value)
1.1. Goal setting (behaviour)	32 (14%)	0.64 (0.21 to 1.96, 0.44)	0.64 (0.16 to 2.54, 0.53)	Goals and planning	0.81 (0.34 to 1.94, 0.64)
1.2. Problem solving	24 (10%)	0.95 (0.31 to 2.95, 0.93)	2.08 (0.49 to 8.81, 0.32)		
1.5. Review behaviour goal(s)	49 (22%)	0.60 (0.24 to 1.54, 0.29)	0.64 (0.22 to 1.85, 0.41)		
2.6. Biofeedback	30 (13%)	0.95 (0.34 to 2.65, 0.92)	1.31 (0.43 to 3.94, 0.63)	Feedback and monitoring	1.34 (0.60 to 3.26, 0.44)
2.7. Feedback on outcome(s) of behaviour future	35 (14%)	0.98 (0.38 to 2.56, 0.97)	0.97 (0.35 to 2.69, 0.95)		
3.2. Social support (practical)	51 (23%)	1.03 (0.45 to 2.34, 0.95)	0.96 (0.40 to 2.26, 0.92)		1.05 (0.45 to 2.45, 0.91)
Instruction on how to perform the behaviour and credible source	35 (16%)	0.76 (0.28 to 2.11, 0.61)	0.70 (0.21 to 2.30, 0.56)		0.73 (0.23 to 2.29, 0.59)
10.4. Social reward	29 (13%)	0.74 (0.24 to 2.25, 0.59)	0.77 (0.23 to 2.60, 0.67)		0.72 (0.21 to 2.40, 0.59)

**Table 5.** CALO-RE behavioural change techniques and their impact on 5% bodyweight loss.

CALORE		Univariable	Multivariable
BCT	N (%)	Odds ratio (95% CI, *P* value)	OR (95% CI, *P* value)
Provide information on consequences of behaviour in general	163 (73%)	1.59 (0.68 to 3.67, 0.28)	1.70 (0.56 to 5.11, 0.35)
Provide information on consequences of behaviour to the individual	110 (49%)	0.88 (0.44 to 1.75, 0.71)	1.57 (0.63 to 3.91, 0.33)
Goal setting behaviour	32 (14%)	0.64 (0.21 to 1.96, 0.44)	0.57 (0.14 to 2.28, 0.43)
Barrier identification/ Problem solving	22 (10%)	0.60 (0.24 to 1.54, 0.29)	2.06 (0.50 to 8.45, 0.32)
Prompt review of behavioural goals	49 (22%)	0.98 (0.38 to 2.56, 0.97)	0.63 (0.23 to 1.78, 0.39)
Review of outcome goals (future)	35 (16%)	1.07 (0.34 to 3.35, 0.91)	1.11 (0.39 to 3.15, 0.84)
Fear arousal	59 (26%)	1.52 (0.72 to 3.20, 0.27)	1.33 (0.59 to 2.96, 0.49)
Informing how to do task	35 (16%)	0.76 (0.28 to 2.11, 0.61)	0.58 (0.17 to 1.98, 0.38)
Plan social support	50 (22%)	1.06 (0.47 to 2.41, 0.89)	1.04 (0.43 to 2.48, 0.94)

OR = Odds ratio, *N* = number, 95% CI = 95% confidence interval.

### Objective 3

*Action at three months*: Of the 17 BCTs we examined, 16 showed no association between the use of a BCT during the brief intervention and the patient taking action on their weight. However, in multivariable analysis, ‘biofeedback’ from v1 of the taxonomy was significantly associated with a decreased odds of taking action (adjusted OR 0.34 (95% CI 0.12–0.98, *p* = 0.05)). No other BCTs were associated with taking action at three months (Tables 6 and 7).

**Table 6.** v1 Behavioural Change Techniques effect on patients taking action at three months.

v1		Univariableanalysis of BCTs	Multivariable analysis of BCTs*	Domain name	Multivariable analysis of domain*
BCT	N (%)	OR (95% CI, P value)	OR (95% CI, P value)		Domain OR (95% CI, P value)
1.1. Goal setting (behaviour)	32 (14%)	1.77 (0.37 to 8.48, 0.48)	1.05 (0.26 to 4.18, 0.94)	Goals and planning	0.93 (0.38 to 2.29, 0.87)
1.2. Problem solving	24 (10%)	2.93 (0.36 to 24.16, 0.32)	0.66 (1.58 to 2.80, 0.94)		
1.5. Review behaviour goal(s)	49 (22%)	0.84 (0.31 to 2.24, 0.73)	0.69 (0.24 to 1.98, 0.49)		
2.6. Biofeedback	30 (13%)	1.17 (0.36 to 3.86, 0.79)	0.34 (0.12 to 0.98, 0.05)	Feedback and monitoring	0.64 (0.26 to 1.59, 0.34)
2.7. Feedback on outcome(s) of behaviour future	35 (14%)	1.63 (0.44 to 6.10, 0.47)	2.06 (0.53 to 7.96, 0.29)		
3.2. Social support (practical)	51 (23%)	0.70 (0.27 to 1.81, 0.46)	0.58 (0.23 to 1.50, 0.26)		0.55 (0.22 to 1.34, 0.19)
Instruction on how to perform the behaviour and credible source	35 (16%)	0.74 (0.21 to 2.55, 0.63)	0.73 (0.23 to 2.33, 0.60)		0.60 (0.21 to 1.72, 0.34)
10.4. Social reward	29 (13%)	1.95 (0.41 to 9.28, 0.40)	2.43 (0.57 to 10.44, 0.23)		1.81 (0.46 to 7.11, 0.39)
(0.41 to 9.28, 0.40)	2.43 (0.57 to 10.44, 0.23)				

OR = Odds Ratio, 95% CI = 95% Confidence Interval, *N* = number. *Adjusted for other BCTs or domains, age, sex, baseline weight and IMD score.

**Table 7.** CALO-RE Behavioural Change Techniques and their impact on patients taking action on their weight at 3 and 12 months.

		Action at 3 months		Action at 12 months	
		Univariable	Multivariable*	Univariable	Multivariable*
BCT	N (%)	OR (95% CI, P value)	OR (95% CI, P value)	OR (95% CI, P value)	OR (95% CI, P value)
Provide information on consequences of behaviour in general	163 (73%)	1.63 (0.70 to 3.79, 0.26)	1.12 (0.30 to 4.16, 0.87)	0.70 (0.30 to 1.66, 0.42)	1.03 (0.30 to 3.58, 0.96)
Provide information on consequences of behaviour to the individual	110 (49%)	0.66 (0.28 to 1.55, 0.35)	1.90 (0.64 to 5.61, 0.24)	1.32 (0.65 to 2.71, 0.44)	2.39 (0.83 to 6.88, 0.11)
Goal setting behaviour	32 (14%)	4.00 (0.50 to 32.16, 0.19)	0.85 (0.22 to 3.33, 0.82)	0.43 (0.17 to 1.10, 0.07)	0.30 (0.08 to 1.09, 0.07)
Prompt review of behavioural goals	22 (10%)	0.83 (0.31 to 2.21, 0.71)	0.64 (0.23 to 1.78, 0.39)	1.02 (0.43 to 2.45, 0.97)	1.85 (0.57 to 6.01, 0.31)
Prompt review of outcome goals (future)	49 (22%)	1.60 (0.43 to 5.98, 0.48)	1.54 (0.40 to 5.89, 0.53)	3.93 (0.89 to 17.38, 0.07)	4.56 (0.93 to 22.27, 0.06)
Barrier identification/ Problem solving	35 (16%)	2.20 (0.26 to 18.68, 0.47)	0.75 (0.19 to 2.99, 0.67)	0.37 (0.14 to 0.97, 0.04**)	0.26 (0.07 to 1.02, 0.05)
Fear arousal	59 (26%)	1.18 (0.40 to 3.51, 0.76)	0.86 (0.33 to 2.25, 0.76)	0.74 (0.34 to 1.62, 0.45)	0.79 (0.32 to 1.96, 0.62)
Informing how to do task	35 (16%)	0.73 (0.21 to 2.52, 0.62)	0.69 (0.21 to 2.25, 0.54)	0.83 (0.33 to 2.12, 0.71)	1.54 (0.44 to 5.46, 0.50)
Plan social support	50 (22%)	0.69 (0.27 to 1.79, 0.45)	0.73 (0.28 to 1.91, 0.52)	0.70 (0.31 to 1.60, 0.40)	0.79 (0.31 to 2.03, 0.63)

OR = odds ratio, 95% CI = 95% confidence interval, *N* = number. *Adjusted for other BCTs or domains, age, sex, baseline weight and IMD score. **p<0.05

*Action at 12 months*: 15 of 17 BCTs showed no association with taking action on weight but ‘problem solving’ from v1of the taxonomy was associated with decreased odds of action (adjusted OR 0.20 (95% CI 0.05–0.81, *p* = 0.02)). ‘Feedback on outcomes of behaviour (future)’ was significantly associated with an increased odds of action (adjusted OR 6.1 (95% CI 1.2–31.0, *p* = 0.03); see Table 8).

**Table 8.** v1 Behavioural Change Techniques and their impact on patients taking action at 12 months.

v1		Univariable analysis of BCTs	Multivariable analysis of BCTs*		Multivariable analysis of domains*
BCT	N (%)	OR (95% CI, *P* value)	OR (95% CI, *P* value)	Domain name	Domain OR (95% CI, *P* value)
1.1. Goal setting (behaviour)	32 (14%)	0.52 (0.21 to 1.25, 0.14)	0.48 (0.14 to 1.67, 0.25)	Goals and planning	1.14 (0.48 to 2.70, 0.76)
1.2. Problem solving	24 (10%)	0.34 (0.14 to 0.87, 0.03**)	0.20 (0.05 to 0.81, 0.02**)		
1.5. Review behaviour goal(s)	49 (22%)	1.02 (0.43 to 2.45, 0.97)	1.99 (0.59 to 6.77, 0.27)		
2.6. Biofeedback	30 (13%)	0.59 (0.22 to 1.54, 0.28)	0.43 (0.14 to 1.36, 0.15)	Feedback and monitoring	1.09 (0.44 to 2.69, 0.85)
2.7. Feedback on outcome(s) of behaviour future	35 (14%)	3.93 (0.89 to 17.38, 0.07)	6.10 (1.20 to 31.0, 0.03**)		
3.2. Social support (practical)	51 (23%)	0.61 (0.27 to 1.38, 0.24)	0.67 (0.27 to 1.69, 0.40)		0.59 (0.25 to 1.39, 0.23)
Instruction on how to perform the behaviour and credible source	35 (16%)	0.83 (0.33 to 2.12, 0.71)	1.76 (0.50 to 6.18, 0.38)		0.77 (0.26 to 2.23, 0.63)
10.4. Social reward	29 (13%)	1.06 (0.37 to 3.02, 0.92)	1.39 (0.39 to 4.96, 0.61)		0.98 (0.31 to 3.08, 0.97)

OR = Odds Ratio, 95% CI = 95% confidence interval, *N* = number. *Adjusted for other BCTs or domains, age, sex, baseline weight and IMD score. **p < 0.05.

### Objective 4

*Number of BCTs*: On average three BCTs were used per intervention but this ranged from 1 to 12. Univariate and multivariate analyses indicated that the number of BCTs used in a consultation were not significantly associated with mean weight loss at 12 months or whether action was taken at 3 or 12 months (see Table 9).

**Table 9.** The number of Behavioural Change Techniques used per consultation and its’ impact on mean weight loss and whether action was taken at 3 and 12 months.

Taxonomy	Mean weight loss		Action 3 months		Action 12 months	
	Univariable	Multivariable*	Univariable	Multivariable*	Univariable	Multivariable*
	Mean weight change kg (95% CI, P value)		OR (95% CI, P value)		OR (95% CI, P value)	
v1	0.35 (−0.19 to 0.89, 0.20)	0.31 (−0.24 to 0.85, 0.27)	0.83 (0.64 to 1.07, 0.15)	0.82 (0.63 to 1.06)	1.07 (0.52 to 2.24, 0.85)	1.13 (0.53 to 2.41)
CALO-RE	0.11 (−0.27 to 0.50, 0.56)	0.12 (−0.29 to 0.52, 0.57)	1.11 (0.86 to 1.44, 0.42)	0.94 (0.78 to 1.14, 0.55)	0.93 (0.79 to 1.09, 0.37)	0.93 (0.78 to 1.11, 0.42)

OR = odds ratio, 95% CI = 95% confidence interval. *Adjusted for age, sex, baseline weight and IMD score.

Sensitivity analyses using GP as a random effects term yielded similar results to the original analyses (see Supplementary Tables S2 and S3).

## Discussion

### Principal findings

In 224 consultations, GPs used an average of three BCTs per consultation to motivate people with obesity to take action on their weight. In total, we were able to analyse 11 unique BCTs, finding that there was little evidence of association. Of those, where there was evidence, the associations were not consistent between taking action on weight and weight change. There was no evidence that the total number of BCTs used in an intervention was associated with weight loss.

### Findings in the context of existing literature

Existing studies have shown associations between the BCT ‘Instruction on how to perform behaviours’ and increased physical activity (Dombrowski et al., 2012; Williams & French, 2011), and improvements to diet. In contrast to this, we found it was associated with weight gain but not with the actions people took to address their weight. In our data, this BCT generally consisted of giving a leaflet or direction to the NHS website. This was often offered after barriers to change had been presented by a patient. Doctors may have noticed people who were less ready or able to change their behaviours and used this BCT as a simple way to intervene without extending the appointment, and it was often used at the end of a consultation. Therefore, it is possible that the association resulted from reverse causation.

The use of the BCT ‘problem solving’ was significantly associated with patients being less likely to have taken action at 12 months. This contrasts with existing studies which identified associations between problem solving and changes to dietary and physical activity behaviours (Avery et al., 2012; Lara et al., 2014). In our data, GPs used problem solving in response to a patient presenting reasons why they could not change their diet or physical activity, such as disability. This again suggests reverse causation. In addition, this intervention was opportunistic and delivered to a group without prior motivation to lose weight, whereas other studies targeted those already motivated to change (Stevens et al., 2003), or those who have multiple meetings to work on motivating behaviour change (Mayer-Davis et al., 2004).

‘Biofeedback’, defined as ‘Quantitative feedback about the body’ (Supplementary Table S4) was significantly associated with patients being less likely to take action at three months. In our study, biofeedback typically meant GPs reflecting on the meaning of patients’ weight, for example by discussing BMI. An existing interview study of patients’ perspectives (Hart et al., 2016), and feedback from our PPI group, indicate this may be because BMI is difficult to understand or interpret. The meaning of ‘BMI’ and how it was calculated, was not always explained in the consultations, which could have led to misunderstandings. An additional consideration is that this result was only significant at three, but not 12 months, and may represent a chance finding.

In the v1 analysis, future follow-up was associated with an increased likelihood of taking action on weight at 12 months. However, the large confidence intervals mean this should be interpreted with great caution. This BCT was typically coded when GPs offered to set up a follow up appointment four weeks later to discuss weight, although we do not know if patients booked or attended these appointments. However, whilst we might expect some actions (such as physical activity) to be associated with maintenance rather than weight loss this was associated with weight gain in our analyses, suggesting that the one significant finding here is spurious. Existing literature highlights that people living with obesity have reported active support and monitoring as important (Ananthakumar et al., 2020). The follow-up provided by this BCT may have supported behaviour change by offering these important elements. Future follow-up in the CALO-RE taxonomy, however, was not significantly associated with patients taking action at 12 months (*p* = 0.06), even though the BCT was defined and coded the same as in the v1 taxonomy. This may be due to the relative effects of the covariates as well as the other BCTs, but the large confidence intervals are consistent with the v1 analysis, and remind us that this should also be interpreted cautiously.

The training video for BWeL clinicians recommended that they use the BCT ‘information about health consequences’. Existing literature shows mixed evidence in the effectiveness of this approach. Dombrowski et al.’s systematic review of the active ingredients in behavioural interventions for adults living with obesity and related co-morbidities, showed that giving information about health consequences does not always motivate weight loss (Dombrowski et al., 2012), and interview studies with people living with obesity also show varied responses to this approach. Whilst some people reported this as helpful information, others found this approach to be fear arousing and unhelpful (Ananthakumar et al., 2020). As ‘information about health consequences’ was used in 220/224 consultations we excluded it from analysis because there was limited comparison. Whilst it is possible that this commonly used BCT could have contributed to the overall weight loss observed in the trial, we were unable to examine this, and would recommend future studies to explore the effectiveness of this BCT further.

It is possible that the reasons BCTs were not associated with outcomes in this context was due to the very brief nature of these intervention. This aligns with a 2020 analyses showing no associations between BCTs used by clinicians when offering very brief opportunistic referral to weight management services, and intervention effectiveness (Bourhill et al., 2021). Brief interventions, as recommended by guidelines, may not allow time for the BCT intensity needed. However, as brief advice was associated with weight loss, irrespective of BCTs used, this should reassure clinicians they can intervene without needing specialist training or knowledge.

### Strengths and limitations

A major strength of our study was that we studied BCTs as they were actually used, rather than simply reported in intervention description or design, and that we related these to empirical outcomes, rather than upstream theoretical or behavioural effects. Another strength is that we used pre-defined codebooks and taxonomies, which allowed us to compare the results of this study with others. The flexibility of these taxonomies also allowed tailoring of the definitions to the study to be more specific to weight loss and the specific style of consultations we listened to. Another strength was the use of multiple coders both developing the codebook and ensuring coding consistency. The decision to use two taxonomies is also a strength as the v1 taxonomy gave breadth, whereas the CALO-RE taxonomy was more tailored towards weight loss behavioural change (Michie et al., 2011). Using two taxonomies allowed for comparison including checking consistent coding of similar BCTs, and the differences allowed a more detailed analysis of certain categories. We also prespecified and date-stamped our analysis plan on Github (Ayre et al., 2019). This gave us a structure to follow throughout the process and aligns with Open Science best practice (Hong & Moran, 2020), and means that all outcomes are reported as described in the analysis plan. Working from audio recordings is also a strength as it prevents participant recall bias. However, whilst we captured intervention delivery in all recordings, in a minority recordings cut off abruptly and there may have been further discussion after the recording cut off in which the GP used more BCTs.

A limitation of our study was weight at three months was self-reported (whilst other weights were objectively measured). We imputed weight following the approaches used in the main trial (Aveyard et al., 2016). Whilst this is considered a conservative approach, a limitation is that some weight data were missing. Another limitation was the low statistical power. This was because of the large number of BCTs used across a relatively small number of recordings. Our sample size of 224 cases allowed detection of an effect size of 1.22 kg weight change with 80% power but the mean weight loss was only 1.04 kg, therefore we may have failed to detect small effects. On the other hand, if the effects of BCTs are so modest as to be undetectable in a cohort that on average successfully lost weight, their clinical relevance is questionable. Our sensitivity analyses using clustering by GP are underpowered (Supplementary Tables S2 and S3), but supported the results. The rate of follow-up at 3 months was lower than at 12 months, and so we used 12-month follow-up data in all our analyses. However, this decision will have led to more noise in our analysis, as other events during the year may have triggered actions with weight change consequences that occurred independently of the GP appointment. Nevertheless, it is long-term weight trajectory that is most relevant for health. Whilst we adjusted for potential confounders (including age, sex, baseline weight and IMD score), it is possible other characteristics which we did not record could have influenced results.

### Clinical implications

Clinicians are encouraged to offer brief advice to patients with obesity, and guidelines offer support in how to do so. Whilst NICE guidelines, for example, recommend using BCTs such as ‘self-monitoring’ and ‘problem solving’ (NICE, 2014b), we found no evidence that these recommended BCTs, or any others, were effective in motivating weight loss. Our results indicate that simply bringing up the topic may encourage weight loss. This is supported by patients reports, which indicate having a GP asking about weight loss efforts and recording weight appears to be very motivating for patients (Ananthakumar et al., 2020).

### Recommendations for further research

Biofeedback was used commonly in consultations and this is missing from the CALO-RE taxonomy. As a taxonomy which is more oriented to weight loss behaviour compared to the v1 taxonomy, the addition of ‘biofeedback’ to the CALO-RE taxonomy could improve the taxonomy’s ability to capture all techniques used in weight loss consultations.

It is common for studies to assess both which individual BCTs were used and the total number employed. However, previous studies have overlooked factors such as where the BCT was used in the consultation. For example, the association of problem solving with weight gain at 12 months may have arisen because this was used in response to patients raising reasons why they could not participate in weight loss. The usual assessment of BCTs takes no account of the method of enactment nor placement or meaning in consultations, which are likely to be key to understanding the impact of communication. Future studies could use methods such as conversation analysis (Barnes, 2005) to capture the potential for different effects depending on the sequence in which actions are delivered (Schegloff, 2007). This has been used to analyse other types of weight management conversations (Albury et al., 2020b), and enabled better identification of effective components of these conversations. Communication about weight management remains an area of ongoing need for clinicians. These data were recorded in 2013–2014, and future studies may wish to examine BCTS used currently in very brief advice to explore if there are differences.

## Conclusion

Clinicians report feeling undertrained to discuss weight loss with patients living with obesity (Alexander et al., 2007; Michie, 2007). This BCT analysis of 224 audio recordings from the BWeL trial found no evidence that including certain BCTs, or certain numbers of BCTs in these conversations increases the effectiveness of very brief advice for weight loss, when there was sufficient power to exclude moderate-sized associations. Taking together all evidence of the trial, this perhaps indicates that it is the brief intervention for weight loss, rather than its content, that motivates weight loss. We found that a brief, simple offer of future follow-up can support people to take action, even if this is not associated with weight loss. Practitioners can feel more confident in offering weight loss advice knowing they do not need complex training to incorporate multiple BCTs, and simply offering follow-up support can motivate change.

## Supplementary Material

Supplemental Material

## Data Availability

This study combines audio recorded consultation data with a de-identified participant dataset from the BWeL trial. Following ethical requirements, audio recorded patient data will not be made available. For information on BWeL trial participant dataset contact Professor Paul Aveyard.

## References

[CIT0001] Agha, M., & Agha, R. (2017). The rising prevalence of obesity: Part A: Impact on public health. *International Journal of Surgical Oncology (NY)*, *2*(7), e17. 10.1097/IJ9.0000000000000017 PMC5673154 29177227

[CIT0002] Albury, C., Strain, W. D., Brocq, S. L., Logue, J., Lloyd, C., & Tahrani, A. (2020a). The importance of language in engagement between health-care professionals and people living with obesity: A joint consensus statement. *The Lancet Diabetes & Endocrinology*, *8*(5), 447–455. 10.1016/S2213-8587(20)30102-9 32333880

[CIT0003] Albury, C. V. A., Ziebland, S., Webb, H., Stokoe, E., & Aveyard, P. (2020b). Discussing weight loss opportunistically and effectively in family practice: A qualitative study of clinical interactions using conversation analysis in UK family practice. *Family Practice*, *38*(3), 321–328. 10.1093/fampra/cmaa121. PMC8211147 33340401

[CIT0004] Alexander, S. C., Ostbye, T., Pollak, K. I., Gradison, M., Bastian, L. A., & Brouwer, R. J. (2007). Physicians’ beliefs about discussing obesity: Results from focus groups. *American Journal of Health Promotion: AJHP*, *21*(6), 498–500. 10.4278/0890-1171-21.6.498 17674636

[CIT0005] Ananthakumar, T., Jones, N. R., Hinton, L., & Aveyard, P. (2020). Clinical encounters about obesity: Systematic review of patients’ perspectives. *Clinical Obesity*, *10*(1), e12347. 10.1111/cob.12347 31793217

[CIT0006] Appel, L. J., Clark, J. M., Yeh, H.-C., Wang, N.-Y., Coughlin, J. W., Daumit, G., Miller, E. R., Dalcin, A., Jerome, G. J., Geller, S., Noronha, G., Pozefsky, T., Charleston, J., Reynolds, J. B., Durkin, N., Rubin, R. R., Louis, T. A., & Brancati, F. L. (2011). Comparative Effectiveness of Weight-Loss Interventions in Clinical Practice. *New England Journal of Medicine*, *365*(21), 1959–1968. 10.1056/NEJMoa1108660 22085317 PMC4074540

[CIT0007] Avery, L., Flynn, D., van Wersch, A., Sniehotta, F. F., & Trenell, M. I. (2012). Changing physical activity behavior in type 2 diabetes: A systematic review and meta-analysis of behavioral interventions. *Diabetes Care*, *35*(12), 2681–2689. 10.2337/dc11-2452 23173137 PMC3507564

[CIT0008] Aveyard, P., Lewis, A., Tearne, S., Hood, K., Christian-Brown, A., Adab, P., Begh, R., Jolly, K., Daley, A., Farley, A., Lycett, D., Nickless, A., Yu, L.-M., Retat, L., Webber, L., Pimpin, L., & Jebb, S. A. (2016). Screening and brief intervention for obesity in primary care: A parallel, two-arm, randomised trial. *The Lancet*, *388*(10059), 2492–2500. 10.1016/S0140-6736(16)31893-1 PMC5121130 27789061

[CIT0009] Ayre, E., Lee, J. J., Frie, K., Aveyard, P., & Albury, C. (2019). Analysis protocol BWeL. https://github.com/grinthreyhound/Ellie_Bwel_analysis_protocol/blob/master/analysisplanEllieAyre.docx.

[CIT0010] Barnes, R. (2005). Conversation analysis: A practical resource in the health care setting. *Medical Education*, *39*(1), 113–115. 10.1111/j.1365-2929.2004.02037.x 15612908

[CIT0011] Blackburn, G. (1995). Effect of degree of weight loss on health benefits. *Obesity Research*, *3*(Suppl 2), 211s–216s. 10.1002/j.1550-8528.1995.tb00466.x 8581779

[CIT0012] Bourhill, J., Lee, J. J., Frie, K., Aveyard, P., & Albury, C. (2021). What makes opportunistic GP interventions effective? An analysis of behavior change techniques used in 237 GP-delivered brief interventions for weight loss. *Annals of Behavioral Medicine*, *55*(3), 228–241. 10.1093/abm/kaaa046 32686819 PMC7980762

[CIT0013] Brauer, P., Gorber, S. C., Shaw, E., Singh, H., Bell, N., Shane, A. R. E., Jaramillo, A., Tonelli, M. (2015). Obesity in adults: Recommendations for prevention of weight gain and use of behavioural and pharmacologic interventions to manage overweight and obesity in adults in primary care. *Canadian Medical Association Journal*, *187*(3), 184–195. 10.1503/cmaj.140887 25623643 PMC4330141

[CIT0014] Brown, I., Thompson, J., Tod, A., & Jones, G. (2006). Primary care support for tackling obesity: A qualitative study of the perceptions of obese patients. *British Journal of General Practice*, *56*(530), 666. https://bjgp.org/content/56/530/666 PMC1876632 16953998

[CIT0015] Brown, J. D., Buscemi, J., Milsom, V., Malcolm, R., & O'Neil, P. M. (2016). Effects on cardiovascular risk factors of weight losses limited to 5-10. *Translational Behavioral Medicine*, *6*(3), 339–346. 10.1007/s13142-015-0353-9 27528523 PMC4987606

[CIT0016] Cradock, K. A., ÓLaighin, G., Finucane, F. M., Gainforth, H. L., Quinlan, L. R., & Ginis, K. A. (2017). Behaviour change techniques targeting both diet and physical activity in type 2 diabetes: A systematic review and meta-analysis. *The international Journal of Behavioral Nutrition and Physical Activity*, *14*(1), 18. 10.1186/s12966-016-0436-0 28178985 PMC5299734

[CIT0017] Dombrowski, S. U., Sniehotta, F. F., Avenell, A., Johnston, M., MacLennan, G., & Araújo-Soares, V. (2012). Identifying active ingredients in complex behavioural interventions for obese adults with obesity-related co-morbidities or additional risk factors for co-morbidities: A systematic review. *Health Psychology Review*, *6*(1), 7–32. 10.1080/17437199.2010.513298

[CIT0018] French, D. P., Olander, E. K., Chisholm, A., & Mc Sharry, J. (2014). Which behaviour change techniques are most effective at increasing older adults’ self-efficacy and physical activity behaviour? A systematic review. *Annals of Behavioral Medicine*, *48*(2), 225–234. 10.1007/s12160-014-9593-z 24648017

[CIT0019] Government efCaL. (2015). The English indices of deprivation 2015 – Frequently asked questions (FAQs).

[CIT0020] Gray, C. M., Hunt, K., Lorimer, K., Anderson, A. S., Benzeval, M., & Wyke, S. (2011). Words matter: A qualitative investigation of which weight status terms are acceptable and motivate weight loss when used by health professionals. *BMC Public Health*, *11*(513). 10.1186/1471-2458-11-513 PMC3142235 21714892

[CIT0021] Greaves, C. J., Sheppard, K. E., Abraham, C., Hardeman, W., Roden, M., Evans, P. H., & Schwarz, P. (2011). Systematic review of reviews of intervention components associated with increased effectiveness in dietary and physical activity interventions. *BMC Public Health*, *11*(1), 119. 10.1186/1471-2458-11-119 21333011 PMC3048531

[CIT0022] Gunther, S., Guo, F., Sinfield, P., Rogers, S., & Baker, R. (2012). Barriers and enablers to managing obesity in general practice: A practical approach for use in implementation activities. *Quality in Primary Care*, *20*(2), 93–103. 22824562

[CIT0023] Hankonen, N., Sutton, S., Prevost, A. T., Simmons, R. K., Griffin, S. J., Kinmonth, A. L., & Hardeman, W. (2014). Which behavior change techniques are associated with changes in physical activity, diet and body mass index in people with recently diagnosed diabetes? *Annals of Behavioral Medicine*, *49*(1), 7–17. 10.1007/s12160-014-9624-9 PMC4335098 24806469

[CIT0024] Hardeman, W., Michie, S., Fanshawe, T., Prevost, A. T., McLoughlin, K., & Kinmonth, A. L. (2008). Fidelity of delivery of a physical activity intervention: Predictors and consequences. *Psychology & Health*, *23*(1), 11–24. 10.1080/08870440701615948 25159904

[CIT0025] Hart, J., Yelland, S., Mallinson, A., Hussain, Z., & Peters, S. (2016). When is it ok to tell patients they are overweight? General public’s views of the role of doctors in supporting patients’ dieting and weight management. *Journal of Health Psychology*, *21*(9), 2098–2107. 10.1177/1359105315571974 25759374

[CIT0026] Hartmann-Boyce, J., Johns, D. J., Jebb, S. A., & Aveyard, P. (2014). Effect of behavioural techniques and delivery mode on effectiveness of weight management: Systematic review, meta-analysis and meta-regression. *Obesity Reviews*, *15*(7), 598–609. 10.1111/obr.12165 24636238 PMC4237119

[CIT0027] Hong, M., & Moran, A. (2020). *An introduction to open science*. https://www.apa.org/science/about/psa/2019/02/open-science.

[CIT0028] Lara, J., Evans, E. H., O'Brien, N., et al. (2014). Association of behaviour change techniques with effectiveness of dietary interventions among adults of retirement age: a systematic review and meta-analysis of randomised controlled trials. *BMC Medicine*, *12*(177). 10.1186//s12916-014-0177-3 PMC4198739 25288375

[CIT0029] Malterud, K., & Ulriksen, K. (2011). Obesity, stigma, and responsibility in health care: A synthesis of qualitative studies. *International Journal of Qualitative Studies on Health and Well-being*, *6*(4), 3402–3404. 10.3402/qhw.v6i4.8404 PMC3223414 22121389

[CIT0030] Mayer-Davis, E. J., D'Antonio, A. M., Smith, S. M., Kirkner, G., Levin Martin, S., Parra-Medina, D., & Schultz, R. (2004). Pounds off with empowerment (POWER): A clinical trial of weight management strategies for black and white adults with diabetes who live in medically underserved rural communities. *American Journal of Public Health*, *94*(10), 1736–1742. 10.2105/AJPH.94.10.1736 15451743 PMC1448527

[CIT0031] Merrill, E., & Grassley, J. (2008). Women's stories of their experiences as overweight patients. *Journal of Advanced Nursing*, *64*(3), 139–146. 10.1111/j.1365-2648.2008.04794.x 18764854

[CIT0032] Michalopoulou, M., Jebb, S. A., & Aveyard, P. (2022). Effectiveness of motivational interviewing in managing overweight and obesity. *Annals of Internal Medicine*, *175*(9), W105. 10.7326/L22-0238 36122404

[CIT0033] Michie, S. (2007). Talking to primary care patients about weight: A study of GPs and practice nurses in the UK. *Psychology, Health & Medicine*, *12*(5), 521–525. 10.1080/13548500701203441 17828672

[CIT0034] Michie, S., Abraham, C., Whittington, C., McAteer, J., & Gupta, S. (2009). Effective techniques in healthy eating and physical activity interventions: A meta-regression. *Health Psychology*, *28*(6), 690–701. 10.1037/a0016136 19916637

[CIT0035] Michie, S., Ashford, S., Sniehotta, F. F., Dombrowski, S. U., Bishop, A., & French, D. P. (2011). A refined taxonomy of behaviour change techniques to help people change their physical activity and healthy eating behaviours: the CALO-RE taxonomy. *Psychology & Health*, *26*(11), 1479–1498. 10.1080/08870446.2010.540664 21678185

[CIT0036] Michie, S., Richardson, M., Johnston, M., Abraham, C., Francis, J., Hardeman, W., Eccles, M. P., Cane, J., & Wood, C. E. (2013). The behavior change technique taxonomy (v1) of 93 hierarchically clustered techniques: Building an international consensus for the reporting of behavior change interventions. *Annals of Behavioral Medicine*, *46*(1), 81–95. 10.1007/s12160-013-9486-6 23512568

[CIT0037] Moyer, V. A. (2012). Screening for and management of obesity in adults: U.S. Preventive Services Task Force recommendation statement. *Annals of internal medicine*, *157*(5), 373. 10.7326/0003-4819-157-5-201209040-00475 22733087

[CIT0038] NICE. (2014a). Overview weight management: Lifestyle services for overweight or obese adults | Guidance.

[CIT0039] NICE. (2014b). Obesity: Guidance on the prevention, identification, assessment and management of overweight and obesity in adults and children. http://www.nice.org.uk/

[CIT0040] Nolan, C., Deehan, A., Wylie, A., & Jones, R. (2012). Practice nurses and obesity: Professional and practice-based factors affecting role adequacy and role legitimacy. *Primary Health Care Research & Development*, *13*(4), 353–363. 10.1017/S1463423612000059 22464138

[CIT0041] Noordman, J., Verhaak, P., & van Dulmen, S. (2010). Discussing patient’s lifestyle choices in the consulting room: Analysis of GP-patient consultations between 1975 and 2008. *BMC family practice*, *11*(87). 10.1186/1471-2296-11-87 PMC2993663 21062427

[CIT0042] Pearson, K. (1896). Mathematical contributions to the theory of evolution. III. Regression, heredity, and panmixia. *Philosophical Transactions of the Royal Society of London Series A, Containing Papers of a Mathematical or Physical Character*, *187*, 253–318.

[CIT0043] Ross, H. M., Laws, R., Reckless, J., Lean, M., McQuigg, M., Laws, R., Noble, P., McCombie, L., Lyons, F., Mongia, S., Quinn, M., Haynes, S., Broom, J. I., Reckless, J., Kumar, S., Lean, M., Frost, G., Barth, J. H., & Nick Finer, D. (2008). Evaluation of the Counterweight Programme for obesity management in primary care: a starting point for continuous improvement. *The British Journal of General Practice*, *58*(553), 548–554. 10.3399/bjgp08X319710 18682018 PMC2486382

[CIT0044] Russell, M. A., Wilson, C., Taylor, C., & Baker, C. D. (1979). Effect of general practitioners’ advice against smoking. *British Medical Journal*, *2*(6184), 231–235. 10.1136/bmj.2.6184.231 476401 PMC1595592

[CIT0045] Schegloff, E. A. (2007). *Sequence organization in interaction: A primer in conversation analysis I*. UK Cambridge University Press.

[CIT0046] Schichtel, M., Wee, B., Perera, R., Onakpoya, I., Albury, C., & Barber, S. (2019). Clinician-targeted interventions to improve advance care planning in heart failure: A systematic review and meta-analysis. *Heart*, *105*(17), 1316–1324. 10.1136/heartjnl-2019-314758 31118199

[CIT0047] Stevens, V. J., Glasgow, R. E., Toobert, D. J., Karanja, N., & Smith, K. S. (2003). One-year results from a brief, computer-assisted intervention to decrease consumption of fat and increase consumption of fruits and vegetables. *Preventive Medicine*, *36*(5), 594–600. 10.1016/S0091-7435(03)00019-7 12689805

[CIT0048] Tate, D. F., Lytle, L. A., Sherwood, N. E., Haire-Joshu, D., Matheson, D., Moore, S. M., Loria, C. M., Pratt, C., Ward, D. S., Belle, S. H., & Michie, S. (2016). Deconstructing interventions: Approaches to studying behavior change techniques across obesity interventions. *Translational Behavioral Medicine*, *6*(2), 236–243. 10.1007/s13142-015-0369-1 27356994 PMC4927444

[CIT0049] Tsiga, E., Panagopoulou, E., Sevdalis, N., Montgomery, A., & Benos, A. (2013). The influence of time pressure on adherence to guidelines in primary care: An experimental study. *BMJ Open*, *3*(4), e002700. 10.1136/bmjopen-2013-002700 PMC3641486 23585394

[CIT0050] Ward, S., Gray, A., & Paranjape, A. (2009). African Americans’ perceptions of physician attempts to address obesity in the primary care setting. *Journal of General Internal Medicine*, *24*(5), 579–584. 10.1007/s11606-009-0922-z 19277791 PMC2669857

[CIT0051] Whitlock, E. P., Orleans, C. T., Pender, N., & Allan, J. (2002). Evaluating primary care behavioral counseling interventions: An evidence-based approach 1 1The full text of this article is available via AJPM Online at www.ajpm-online.net. *American Journal of Preventive Medicine*, *22*(4), 267–284. 10.1016/S0749-3797(02)00415-4 11988383

[CIT0052] WHO. (2015). WHO overview – Preventing chronic diseases: A vital investment.

[CIT0053] Williams, S. L., & French, D. P. (2011). What are the most effective intervention techniques for changing physical activity self-efficacy and physical activity behaviour – and are they the same? *Health Education Research*, *26*(2), 308–322. 10.1093/her/cyr005 21321008

