# Supplementary material for: GP delivered brief weight loss advice: associations between in-consultation behaviour change techniques and patient weight loss in recorded primary care discussions
Source: Health Psychol Behav Med. 2023 May 20;11(1):2213751. doi: 10.1080/21642850.2023.2213751 (PMC10201997; doi:10.1080/21642850.2023.2213751)
Supplement: Supplemental Material [file RHPB_A_2213751_SM7758.docx]

|  | **Frequency** | **Percentage of consultations where BCT was used** |
| --- | --- | --- |
| **BCT Taxonomy V1** | | |
| - Information about health consequences | 220 | 98 |
| - Social support (practical) | 51 | 23 |
| - Review behaviour goal(s) | 49 | 22 |
| - Feedback on outcome(s) of behaviour | 35 | 16 |
| - Instruction on how to perform the behaviour and credible source | 35 | 16 |
| - Goal setting (behaviour) | 32 | 14 |
| - Biofeedback | 30 | 13 |
| - Social reward | 29 | 13 |
| - Problem solving | 24 | 11 |
| **CALO-RE Taxonomy** | | |
| - Provide information on consequences of behaviour in general | 163 | 73 |
| - Provide information on consequences of behaviour to the individual | 110 | 49 |
| - Fear arousal | 59 | 26 |
| - Plan social support | 50 | 22 |
| - Prompt review of behavioural goals | 49 | 22 |
| - Prompt review of outcome goals (future) | 35 | 16 |
| - Instructing on when and where to perform behaviour | 35 | 16 |
| - Goal setting behaviour | 32 | 14 |
| - Barrier identification/Problem solving | 22 | 10 |

Supplementary Table 1: BCTs used in >20 consultations

| v1 |  | Multivariable analysis of BCTs* |  | Multivariable analysis of domains* |
| --- | --- | --- | --- | --- |
| BCT | N (%) | Mean weight change in Kg (95% CI, P value) | Domain name | Mean weight change in Kg (95% CI, P value) |
| 1.1.  Goal setting (behaviour) | 32 (14%) | -0.85 (-3.46 to 1.76, 0.52) | Goals and planning | -0.22 (-2.02 to 1.57, 0.81) |
| 1.2.  Problem solving | 24 (10%) | -0.92 (-3.47 to 1.76) |  |  |
| 1.5.  Review behaviour goal(s) | 49 (22%) | 1.05 (-1.00 to 3.09, 0.32) |  |  |
| 2.6. Biofeedback | 30 (13%) | -1.24 (-3.54 to 1.05, 0.29) | Feedback and monitoring | -1.12 (-4.04 to 1.21, 0.29) |
| 2.7. Feedback on outcome(s) of behaviour future | 35 (14%) | 1.93 (-0.20 to 4.06, 0.08) |  |  |
| 3.2. Social support (practical) | 51 (23%) | -0.29 (-2.12 to 1.55, 0.76) | Social Support | -0.34 (-2.15 to 1.48, 0.72) |
| 4.1 and 9.1 Instruction on how to perform the behaviour and credible source | 35 (16%) | 2.53 (0.19 to 4.87, 0.03**) | Shaping Knowledge/Comparison of outcomes | 2.39 (0.13 to 4.65, 0.04**) |
| 10.4. Social reward | 29 (13%) | -0.47 (-2.85 to 1.91, 0.70) | Reward and Threat | -0.19 (-2.58 to 2.19, 0.87) |

*Supplementary table 2: sensitivity analysis using mixed effects model, with GP practice as a*

*random effects term. Associations with mean weight change, v1. CI= confidence interval. *adjusted for other BCTs or domains, age, sex, baseline weight and IMD score.**p<0.05*

| **BCT** | **Mean weight loss (kg) (95% CI, P value)** |
| --- | --- |
| Information provision general | 0.002 (-2.38 to 2.38, 0.999) |
| Information provision individual | -0.64 (-2.68 to 1.41. 0.54) |
| Goal setting behaviour | -0.26 (-3.20 to 2.68, 0.86) |
| Review of behavioural goals | 1.45 (-0.72 to 3.63, 0.19) |
| Review of outcome goals (future) | 1.57 (-0.69 to 3.83, 0.17) |
| Problem solving | -1.32 (-4.43 to 1.79, 0.41) |
| Fear arousal | -1.07 (-2.89 to 0.75, 0.25) |
| Informing how to do task | 2.47 (0.02 to 4.92, 0.05) |
| Plan social support | -0.18 (-2.09 to 1.72, 0.85) |

*Supplementary table 3: Sensitivity analysis using a mixed effects model: with GP practice as a random effects term. Associations with mean weight change, CALO-RE. CI= confidence interval . adjusted for other BCTs, age, sex, baseline weight and IMD score.*

| **v1 Codebook** | |  |
| --- | --- | --- |
| **BCT used v1 taxonomy** | Definition | Example |
| 1.1.  Goal setting (behaviour) | Set/agree on a goal defined in terms of the behaviour to be achieved. | “Maybe try reducing your portion size by 10%, and eating 5 pieces of fruit and/or vegetables a day” |
| 1.2.  Problem solving | Analyse behaviour and how the patient can overcome barriers to behaviour change. | “So if you can’t do much exercise try cutting down on the snacks… it’s all about balance…” |
| 1.3.  Goal setting (outcome) | Set a goal that the wanted behaviour will have positive effect on, such as the suggestion of a specific amount of weight loss the patient should aim for | “For your ideal weight I want you to try and lose a couple of stone” |
| 1.4.  Action planning | Detailed planning of behaviour: when, what, how, where, time, intensity, if not code 1.1 | “If you go walking four times a week for about 45 minutes, a brisk walk, in the mornings with the dog, that’ll help” |
| 1.5.  Review behaviour goal(s) | Review and possibly modify the patients’ behaviour depending on how close/ far from goal they are | “So talk me through what you’d usually eat in a day” |
| 1.7.  Review outcome goal(s) past | Review and provide feedback on previous weight change. | “I see you’ve gained x amount of weight since I last saw you” |
| 2.3. Self-monitoring of behaviour | Establish way the patient can record behaviour | “you could try a food diary, so you write down what you eat in the day, say for a couple of weeks” |
| 2.4. Self-monitoring of outcomes of behaviour | Establish way the patient can record outcomes of behaviour such as the patient weighing themselves | “The best way to do it is to weigh yourself in the morning and make a note of it” |
| 2.6. Biofeedback | Quantitative feedback about the body e.g. BMI, weight etc | “It says here you have a BMI of 35, which we would class as overweight” |
| 2.7. Feedback on outcome(s) of behaviour future | Plan to provide feedback on the outcome of behaviour (weight) in the future usually through setting up another appointment in a specific time frame | “I’d like to set up another appointment with you in a months’ time to reweigh you and see if we need to reassess what we are doing” |
| 3.1. Social support (unspecified) | Advise, arrange or provide social support | “It’s easier if you all eat the same food together, all the family” |
| 3.2. Social support (practical) | The GP provides practical social support | “we can set you up a nurse’s appointment for two weeks’ time and she can go into more detail with you if you’d like?” |
| 3.3. Social support (emotional) | The GP offers emotional support | Generally consoling an upset patient |
| 4.1. Instruction on how to perform the behaviour | Giving advice on how to perform behaviour, including skills training, provision of a leaflet etc | “So here’s a leaflet, it has ideas for healthy meals and exercise and things so see how you go with that” |
| 5.1. Information about health consequences | Written/verbal/visual information about health consequences of performing/not performing the behaviour | “losing weight will decrease your risk of developing things like diabetes and high blood pressure” |
| 5.2. Salience of consequences | Methods such as analogies are used to emphasise consequences of performing/not performing the behaviour to make the information more memorable | “It’s like carrying bags of flour round with you all day, imagine that, wouldn’t be good for your joints…” |
| 5.3 information about evidence base | Some kind of credibility is given to the information being relayed | Saying “studies show…” or “evidence shows us that...” |
| 6.3. Information about others’ approval | Give information about what other people think about wanted/unwanted behaviour | “X/y would like it if you lost a bit of weight, I’m sure” |
| 8.2. Behaviour substitution | Encourage patient to substitute wanted behaviour in for unwanted behaviour | Swapping steak for grilled chicken, cycling to work rather than driving etc |
| 9.1. Credible source | Present communication from a credible source | “Here’s a British heart foundation leaflet, these are really helpful I think…” |
| 9.3. Comparative imagining of future outcomes | Encourage the patient to imagine and compare the future versus now of changed versus unchanged behaviour | “If you don’t exercise mobility will worsen but if you do exercise and lose weight your mobility will get better” |
| 10.4. Social reward | Verbal/non-verbal reward of progress from the GP when the patient performs the wanted behaviours and/or has lost weight | “You’ve lost a stone since last year and that’s great!” |
| 15.3.  Focus on past success | Using reflections on successful past weight loss to increase motivation | “Last year you lost a stone so we know you can do it” |

*Supplementary table 4: Our codebook based on the v1 taxonomy.*

| CALO-RE codebook | |  |
| --- | --- | --- |
| BCT name | Definition | Example |
| Provide information on consequences of behaviour in general | General written/verbal/visual information about health consequences of performing/not performing the behaviour | “Losing weight will reduce your change of developing high blood pressure” |
| Provide information on consequences of behaviour to the individual | Information about benefits/costs of action/inaction to the individual based on individuals’ situation | “I know you’ve got a family history of diabetes so losing a bit of weight could help you avoid that” |
| Information about evidence base | Some kind of credibility is given to the information being relayed | “Studies have shown that losing weight decreases your risk of xyz” |
| Provide information about others approval | Information about what other people think about behaviour: likes, approval or disapproval | “X/y would like it if you lost a bit of weight, I’m sure” |
| Goal setting behaviour | Make a behavioural resolution e.g. instigating or maintaining change | “Try and eat 5 pieces of fruit and veg a day” |
| Goal setting outcome | General goal achieved by behaviour but not defined in terms of behaviour e.g. weight loss | “Aim for losing around a stone” |
| Prompt review of behavioural goals | Opportunity to review of the patients’ current behaviour, possibly modifying behaviour to reach goal | “So talk me through what you’d usually eat in a day” |
| Prompt review of outcome goals (future) | Plan to review patient performance/ outcomes in a specific timeframe | “I’d like to set up another appointment with you in a months’ time to reweigh you and see how you’re doing from there and if we need to reassess what we are doing” |
| Action planning | Detailed planning of what the person will do including when, in which situation and/or where to act. | “If you go walking four times a week for about 45 minutes, a brisk walk, in the mornings with the dog, that’ll help” |
| Barrier identification/ Problem solving | Think about potential barriers to behaviour change and identify the ways of overcoming them | “So if you can’t do much exercise try cutting down on the snacks… it’s all about balance…” |
| Effort/progress contingent rewards | Praise or rewards for attempts at achieving a behavioural goal (material or social) | “Well done for cutting out snacking between lunch and dinner” |
| Successful behaviour contingent rewards | Reinforcing successful performance of the specific target behaviour via material or social reward e.g. praise patient for losing weight | “You’ve lost a stone since last year which is great!” |
| Fear arousal | Present risk/mortality information relevant to the behaviour, such as the risks of not losing weight. Code with information giving general or individual depending whether is generally or individually aimed | “You are at high risk for things like arthritis, diabetes and even certain kinds of cancer” |
| Prompt Self-monitoring behaviour | Keep a record of specified behaviour(s) as a method for changing behaviour | “You could try a food diary, so you write down what you eat in the day, say for a couple of weeks” |
| Prompt Self-monitoring of outcomes | Record of specified measures expected to be influenced by behaviour change | “The best way to do it is to weigh yourself in the morning and make a note of it” |
| Provide Feedback on performance (past) | Providing the participant with information on recent behavioural outcomes to motivate behaviour | “I see you have lost some weight since we last saw you” |
| Informing how to do task | Offer advice/ideas on how to perform behaviour | Usually a leaflet is given or told to look at the NHS website |
| Plan social support | Prompting person to plan how to get support from others to help achieve target behaviour/outcome | “We can set you up a nurse’s appointment for two weeks’ time and she can go into more detail with you if you’d like?” |
| Focus on past success | Instructing the person to think about or list previous successes in performing the behaviour to encourage them to try again/carry on | “So remember when you lost weight a few years back, how great that felt?” |
| Motivational interviewing | Prompting person to engage in change talk, minimise resistance and resolve ambivalence to change. The consultation may be more focused on the patient and why they want to change their behaviour rather than the GPs reasons for change. | “So what is making you want to lose the weight?” |

*Supplementary table 5: Our codebook based on the CALO-RE taxonomy*
